# Leptin as a Biomarker of Stress: A Systematic Review and Meta-Analysis

**DOI:** 10.3390/nu13103350

**Published:** 2021-09-24

**Authors:** Jean-Baptiste Bouillon-Minois, Marion Trousselard, David Thivel, Amanda C. Benson, Jeannot Schmidt, Farès Moustafa, Damien Bouvier, Frédéric Dutheil

**Affiliations:** 1 Université Clermont Auvergne, CNRS, LaPSCo, Physiological and Psychosocial Stress, F-63000 Clermont-Ferrand, France; jschmidt@chu-clermontferrand.fr (J.S.); fdutheil@chu-clermontferrand.fr (F.D.); 2 Emergency Medicine, CHU Clermont-Ferrand, F-63000 Clermont-Ferrand, France; fmoustafa@chu-clermontferrand.fr; 3 Neurophysiology of Stress, Neuroscience and Operational Constraint Department, French Armed Forces Biomedical Research Institute, IRBA, F-91223 Brétigny-sur-Orge, France; marion.trousselard@gmail.com; 4 Université Clermont Auvergne, Laboratory of the Metabolic Adaptations to Exercise under Physiological and Pathological Conditions [AME2P], F-63000 Clermont-Ferrand, France; david.thivel@uca.fr; 5 Sport Innovation Research Group, Department of Health and Biostatistics, Swinburne University of Technology, Melbourne, VIC 3122, Australia; abenson@swin.edu.au; 6 Université Clermont Auvergne, CNRS, INSERM, GReD, F-63000 Clermont-Ferrand, France; dbouvier@chu-clermontferrand.fr; 7 Biochemistry and Molecular Genetic Department, CHU Clermont-Ferrand, F-63000 Clermont-Ferrand, France; 8 Occupational and Environmental Medicine, CHU Clermont-Ferrand, WittyFit, F-63000 Clermont-Ferrand, France

**Keywords:** anxiety, appetite, mental health, metabolism, public health

## Abstract

Background: Leptin is a satiety hormone mainly produced by white adipose tissue. Decreasing levels have been described following acute stress. Objective: To conduct a systematic review and meta-analysis to determine if leptin can be a biomarker of stress, with levels decreasing following acute stress. Methods: PubMed, Cochrane Library, Embase, and ScienceDirect were searched to obtain all articles studying leptin levels after acute stress on 15 February 2021. We included articles reporting leptin levels before and after acute stress (physical or psychological) and conducted random effects meta-analysis (DerSimonian and Laird approach). We conducted Meta-regressions and sensitivity analyses after exclusion of groups outside the metafunnel. Results: We included seven articles—four cohort and three case-control studies—(28 groups) from 27,983 putative articles. Leptin levels decreased after the stress intervention (effect size = −0.34, 95%CI −0.66 to −0.02) compared with baseline levels, with a greater decrease after 60 min compared to mean decrease (−0.45, −0.89 to −0.01) and in normal weight compared to overweight individuals (−0.79, −1.38 to −0.21). There was no difference in the overweight population. Sensitivity analyses demonstrated similar results. Levels of leptin after stress decreased with sex ratio—i.e., number of men/women—(−0.924, 95%CI −1.58 to −0.27) and increased with the baseline levels of leptin (0.039, 0.01 to 0.07). Conclusions: Leptin is a biomarker of stress, with a decrease following acute stress. Normal-weight individuals and women also have a higher variation of leptin levels after stress, suggesting that leptin may have implications in obesity development in response to stress in a sex-dependent manner.

## 1. Introduction

Psychosocial stress is a significant public health concern [1,2,3] with substantial consequences, such as burnout [4], anxiety [5], depression [6], and suicide [7]. Stressed people also have more cardiovascular diseases [8], eating disorders [9], and are more overweighted or obese [10]. Identifying stressful events is an important research topic in medicine because it can lead to policies for prevention. Although the literature has proposed many biomarkers of stress [11,12,13], none is ubiquity, and the majority of those depend on the hypothalamic–pituitary–adrenal axis [14]. Leptin is a 167-aminoacid peptide mainly expressed in white adipose tissue (WAT) [15]. Its discovery changed the view of WAT from a superficial tissue responsible for energy deposit to an active endocrine organ [16]. Leptin is commonly known as a satiety hormone produced by WAT with low levels during the meal initiation and high levels after the meal [15]. Leptin blood levels are not the same in all individuals. Indeed, normal-weight people and men seem to have lower levels of leptin [17,18]. Besides the satiety effect, leptin plays an active role in energy homeostasis [19], metabolism [20], exercise [21], and neuroendocrine function [20]. This neuroendocrine function is centralized by receptor of leptin in the hypothalamic. Low leptin levels contribute to of hyperphagia, hypogonadotropic hypogonadism, and suppression of thyroid and growth hormone (GH) levels [20]. Leptin seems to also have an important impact on glucose homeostasis. Indeed, it seems to affect peripheral insulin sensitivity via central nervous system mechanisms independent of its effects on food intake and weight [20].

Furthermore, it has been proposed to be a biomarker of acute stress, decreasing its levels after acute stress. In physiological conditions, leptin has a short-term half-life—30 min—with 18% daily circadian variations [22,23]. To our knowledge, no meta-analysis to date has examined the effects of acute stress on leptin levels. Secondly, a strong link is present between stress and obesity because of destructive eating behaviors [24].

Thus, we aimed to conduct a systematic review and meta-analysis to determine if leptin levels could be a relevant biomarker of acute stress, with a decrease following the acute stress, and compare the response in leptin levels between overweight and normal-weight individuals.

## 2. Materials and Methods

### 2.1. Literature Search

**Figure 1:**
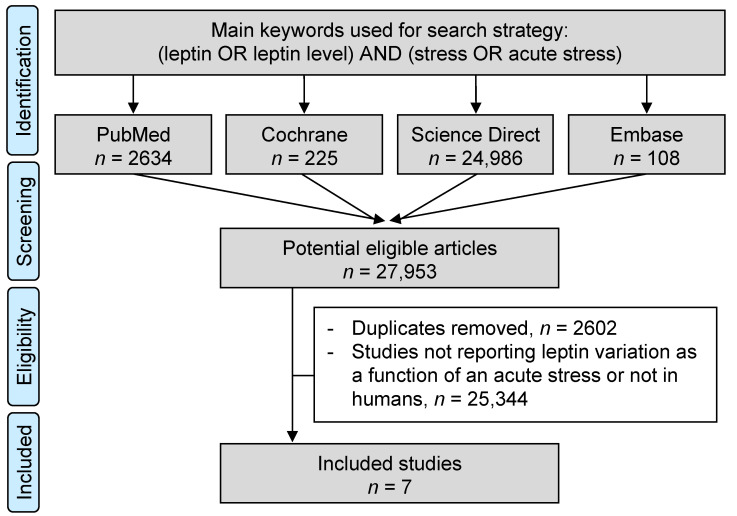
Search strategy.

PubMed, Cochrane Library, Embase, and ScienceDirect databases were searched on 15 February 2021, with the following keywords “leptin” OR “ob protein” OR “ob gene product” OR “obesity factor” OR “obese gene product” OR “obese protein” AND “anxiety” OR “anxious” OR “stress” OR “mood” OR “emotion” (detailed search strategy, Appendix A). The use of broad keywords aimed to be as exhaustive as possible. Articles needed to describe our primary outcome variable, i.e., the measurement of leptin levels before and after acute stress with or without a control group. We did not limit our search to specific years, languages, or regions, but studies on animals and patients with psychiatric disorders were excluded. Reference lists from reviews and articles retrieved from our search were explored to identify any further studies. Two authors (JBB and MT) conducted searches, collated and separately reviewed the abstracts, and decided the suitability of the articles for inclusion. A third author (FD) reviewed the articles where consensus on appropriateness was debated. All authors then examined the eligible articles. The search strategy is presented in Figure 1.

### 2.2. Data Collection

The data collected included first author’s name, publication year, study design, country, aims and outcomes of included articles, sample size, age, sex, characteristics of stress (type, duration), characteristics of measurement (time of measurement, type of fluid, technic of measurement), leptin levels, and putative adjustment/explaining factors (such as body mass index (BMI), insulinoresistance, smoking, or leisure physical activity).

### 2.3. Quality of Assessment

The Newcastle Ottawa Scale (NOS) for cohort studies and the NOS for case-control studies were used to assess the quality of the included articles based on three types of bias (selection, comparability, and exposure) [25]. The maximal score was eight—one point per question.

### 2.4. Statistical Consideration

We used Stata software (version 16, StataCorp, College Station, TX, USA) to conduct the statistical analysis. Random effects meta-analyses (DerSimonian and Laird approach) were conducted on leptin levels before and after acute stress [26]. Results were expressed as effect size (ES), with a negative ES denoting a decrease in leptin levels. An ES at 0.8 reflects a large effect, 0.5 a moderate effect, and 0.2 a small effect. Specifically, we conducted meta-analysis stratified on time after the stress and a meta-analysis stratified by weight—overweight (BMI > 25) versus normal-weight people considering number of articles in each group. We stratified all meta-analysis by time after stress. We determined the stratification time to every 30 min according to the leptin half-life (25 ± 5 min). Sensitivity analyses were conducted after excluding studies that were not evenly distributed around the base of the funnel-plot and to search for potential publication bias. Heterogeneity between studies was also evaluated using I^2^ statistic. I^2^ is easily interpretable with values ranging between 0% and 100%: I^2^ < 25% reflects a low heterogeneity, 25 < I^2^ < 50% reflects a modest heterogeneity, and I^2^ > 50% reflects a high heterogeneity. We also conducted a meta-analysis by type of stress when feasible.

We also conducted meta-regression analyses to explore the influence of putative explaining factors, such as age, sex of participants, BMI and other sociodemographic variables, characteristics of the acute stress (duration, type of stress), and time of sampling. We expressed results as regression coefficients and 95% CI. *p*-value less than 0.05 were considered statistically significant.

## 3. Results

The initial search retrieved 27,983 putative articles (108 in the Cochrane Library, 2634 in PubMed, 108 in Embase, and 24,986 in ScienceDirect). Removal of duplicates and use of the selection criteria—i.e., human study, at least one measure before acute stress and one after the end of the stressful event, approval of an Institutional Review Board or an Ethic Committee—reduced the number of articles reporting the evaluation of leptin level in the blood to seven articles [27,28,29,30,31,32,33] for the systematic review and meta-analysis (Figure 1). All articles were written in English.

### 3.1. Quality of Articles and Study Designs

**Figure 2:**
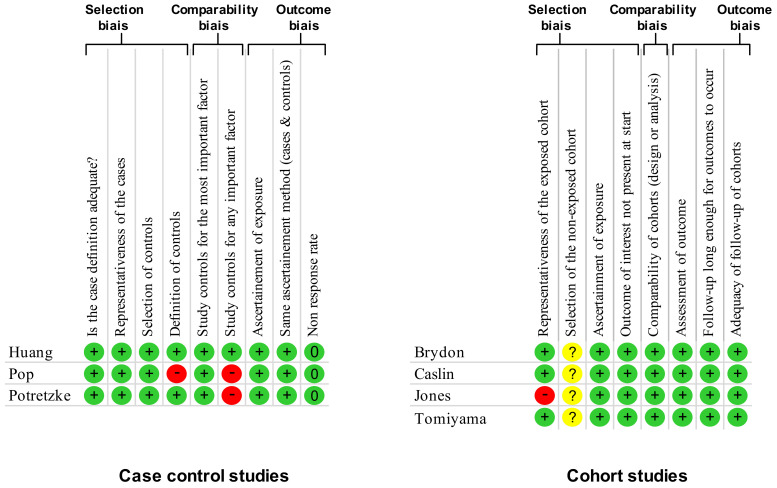
Methodological quality of included articles using Newcastle—Ottawa Quality Assessment Scale Yes, +; No, −; Cannot say, ?; Not applicable, N/A; 0, percentage of non-response.

Using the NOS criteria, all the seven included studies had a score of >6 and were considered high quality (Figure 2). Three studies were case-control studies [29,32,33] without blind assessment, and four were cohort studies [27,28,30,31]. All studies mentioned an ethical approval.

### 3.2. Inclusion and Exclusion Criteria of Included Articles

All participants were adults and were considered “healthy” by investigators, i.e., without any current infectious diseases or a history of autoimmune, endocrine (with a particular mention of diabetes), inflammatory, or neurological disorders. Furthermore, women were not pregnant and could not be lactating. Participants were excluded if they took medication that could interfere with leptin, such as corticosteroids, antibiotics, or anti-inflammatories. The studies were performed in three countries: the United Kingdom [27], the United States of America [28,29,30,31,33], and Romania [32]. Two studies recruited only healthy women (one included all ages [27] and the second only postmenopausal [30]). Two studies recruited healthy normal and overweight men [28,29]. One study recruited healthy smokers who were asked to be abstainers for four weeks [33]. If they succeeded, they were classified as abstainers but were classified as relapsers if they failed. Two studies recruited healthy volunteers comparing normal-weight and overweight individuals in one study [32] and insulin-resistant and insulin-sensitive ones in the other [31]. The separation between insulin-resistant or insulin-sensitive individuals was made after a quantitative insulin-sensitivity check index [34] (QUICKI) with a cut-off point of <0.33 to have insulin resistance and >0.33 to have normal insulin sensitivity (Table 1).

### 3.3. Population

We included a total of 322 participants, and the sample size ranged from 20 [28,29] to 79 [32]. Mean age ranged between 21.2 ± 0.8 [28] to 62.0 ± 6.0 years [30]. Sex was reported in all studies. Three studies recruited only women [27,30,31], two only men [27,28], and two both sexes [32,33]. We included a total of 104 men (32.3%) and 218 women (67.7%). All studies reported body mass index (BMI), ranging from 22 kg/m^2^ [28] to 38 kg/m^2^ [29]. Three studies used BMI to create groups, i.e., normal weight and overweight [27,29,32]. One study used the capacity to abstain from smoking for four weeks to create groups, i.e., abstainers and relapsers [33]. Only two studies reported height and weight [28,29] and only three the percentage of body fat [26,27,28]. Only one study reported smoking [27], waist circumference [27], leisure physical activity [33], HbA1c [27], and insulin resistance [31] and glycemia, LDL, HDL, and triglyceride levels [32].

### 3.4. Main Outcome of Studies

The main outcome of all studies included in our meta-analysis was the effect of acute stress on the variation of leptin blood levels (and possibly other hormones, such as cortisol [27,28,29,30,31,33], IL-6 [27,28,29,31], IL-1Ra [27,28], insulin [31], LDL-HDL [32], and adiponectin [31,32]).

### 3.5. Type of Stress

All studies described the procedure of the stressful event. Six studies used mental stress and one physical stress. Three studies [27,30,31] used the Tried Social Stress Test—a validated tool to provoke psychobiological stress responses composed of a combination which consists of a 3-min preparation period, a 5-min free speech, and a 5-min mental arithmetic task in front of 2–3 audience members. Two studies used a computer-based mental task previously validated as stressful [35]. Patients were presented with a colour-word on the screen, which was written in a different colour font. Simultaneously, the computer said a third colour, while the subjects were required to identify the font colour in which the word was presented. A new colour-word was given every second for 2 min. Following the Stroop colour-word task, subjects were presented with a three-digit number from which they were required to randomly subtract either 3, 7, 8, or 13. Auditory feedback was given by the program when participants entered an incorrect answer. The mental arithmetic task continued for 2 min. This 4-min cycle of two stressors occurred five times for a total of 20 min. Prior to the start of the task, subjects were instructed to work as accurately and quickly as possible and informed that their scores would be recorded. In addition, an investigator stayed in the room. [28,29]. One study used only public speaking. The public speaking challenge entailed participants composing and delivering three 4-min speeches focusing on three scenarios. The three scenarios were presented in a counterbalanced order. In one scenario, participants were presented with a controversial social issue and were asked to introduce their positions and defend them. In another scenario, participants were given an article about an issue of general interest and were asked to construct a presentation based on this article. In a third scenario, participants were asked to imagine a hypothetical situation where they were being accused of shoplifting [33]. The last one used physical stress on a cycle ergometer during the time needed to obtain VO_2_ max. All participants were submitted to symptom-limited, maximal stress testing on a cycloergometer, according to classical protocols. Mean effort was 103 ± 27 Ws, and mean duration 12.5 ± 3 min [32].

### 3.6. Time of Procedure

Three studies were performed during the beginning of the morning—between 7:30 a.m. and 8:30 a.m. [28,29,30]—and three during the beginning of the afternoon—between noon and 2:30 p.m. [27,31,33]. One did not mention when the study was performed [36].

### 3.7. Method and Time of Sampling

All studies measured leptin levels in blood. All studies included except one [32] gave details on the method of sampling and analysis. Blood samples were collected in EDTA-treated tubes and immediately centrifuged to yield plasma for hormone determinations. Concentrations were determined by radio-immuno-assay (RIA) [30,31] or enzyme-linked-immunosorbent-assay (ELISA) [27,28,29,32,33]. Three studies also reported inter-assay and intra-assay coefficient, ranging from 5% [28] to 12% [27] and from 6.6% [30] to 10% [27,28], respectively. All studies measured leptin levels within the two hours following the end of the acute stress.

### 3.8. Meta-Analysis of Leptin Variation

**Figure 3:**
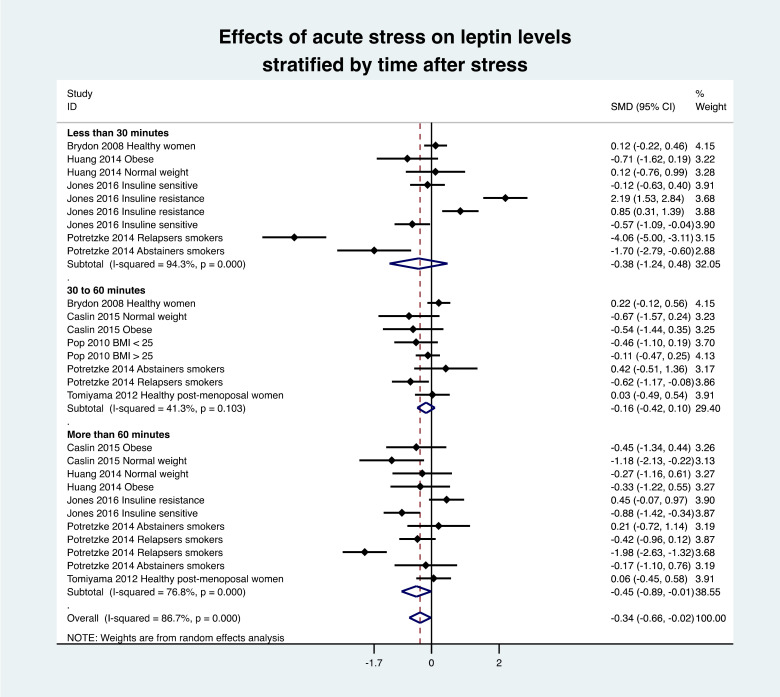
Meta-analysis of leptin levels following acute stress compared to baseline. SMD, standardized mean difference.

**Figure 4:**
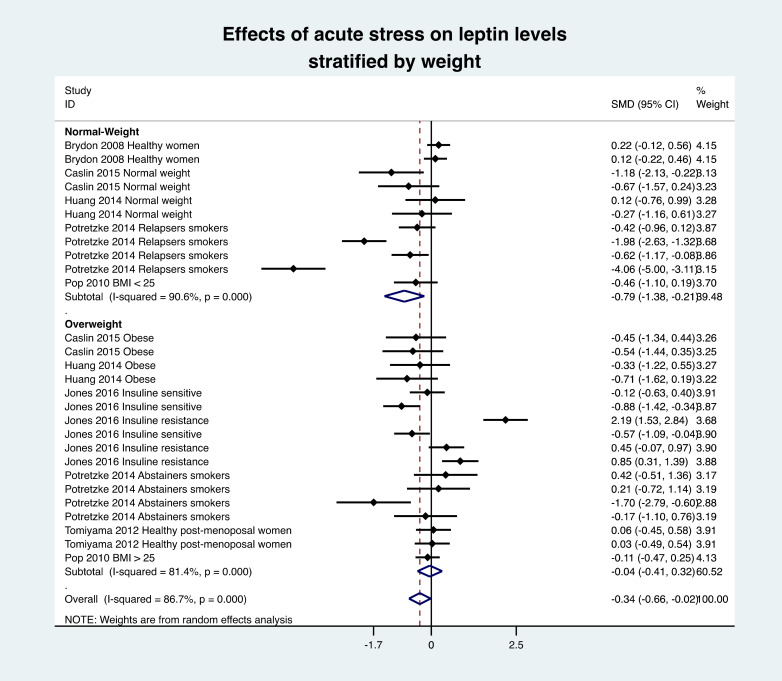
Meta-analysis of leptin levels following acute stress compared to baseline levels, stratified by weight of individuals. SMD, standardized mean difference.

The overall meta-analysis of seven studies (28 groups) demonstrated a decrease in leptin levels after the stress intervention (effect size = −0.34, 95% CI −0.66 to −0.02, I^2^ 86.7%) compared with the baseline levels. Stratification by time showed a non-statistically significant decrease in leptin levels in the 30 min (−0.38, −1.24 to 0.48) and between 30 and 60 min following the stress intervention (−0.16, −0.42 to 0.10) as well as a significant decrease after 60 min (−0.45, −0.89 to −0.01) (Figure 3). Stratification by weight classification showed a significant decrease in leptin levels in the normal-weight population, i.e., BMI < 25 kg/m^2^ (−0.79, −1.38 to −0.21). There was no difference in the overweight population, i.e., BMI > 25 kg/m^2^ (−0.04, −0.41 to 0.32) (Figure 4). Stratification by type of stress was not salient for physical activity, as it was only reported in one study. There was a tendency (*p* = 0.054) for a decrease in leptin levels following an acute mental stress (−0.35, −0.70 to 0.01). This could be explained by the low number of studies (*n* = 6 after exclusion), which increase the 95% confidence interval from −0.66; −0.02 to −0.70; 0.01).

### 3.9. Metafunnel and Meta-Analysis after Exclusion of Studies Outside of the Metafunnel

**Figure 5:**
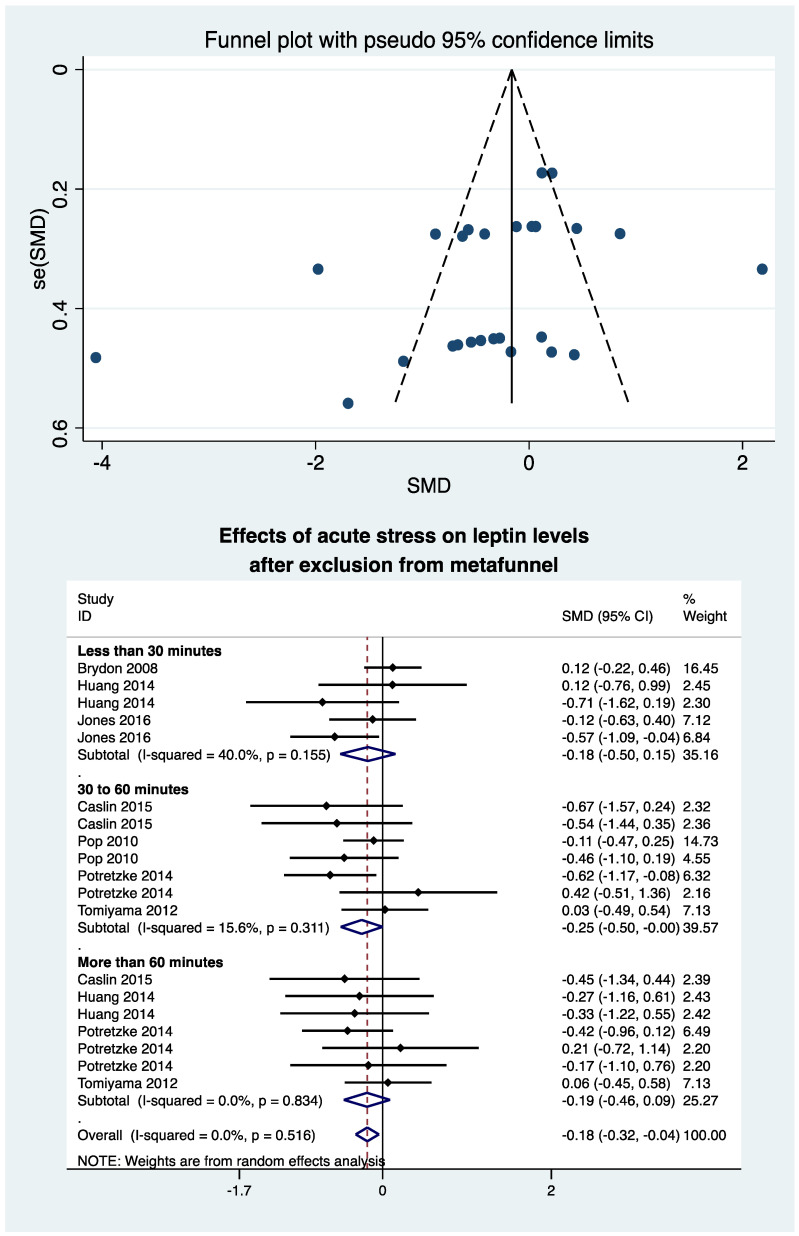
Meta-analysis of leptin levels after acute stress compared to baseline levels after exclusion of studies outside of the metafunnel. SMD, standardized mean difference.

We produced a metafunnel of the seven studies (28 groups) that showed eight groups outside. Meta-analysis after the exclusion of those groups showed similar results, i.e., an overall decrease in leptin levels (ES = −0.18, 95CI −0.32 to −0.04; *p* = 0.010). After exclusion from metafunnel, stratification by time showed a significant decrease between 30 and 60 min (−0.25, −0.50 to −0.00; *p* = 0.049) but a non-significant decrease in the first 30 min (−0.18, −0.50 to 0.15; *p* = 0.29) and after 60 min (−0.19, −0.32 to 0.09; *p* = 0.18) (Figure 5), and there were a tendency for a decrease in leptin levels following an acute stress in 12 groups of normal-weight individuals (−0.27, −0.54 to 0.01; *p* = 0.056) and in seven overweight individuals groups (−0.16, −0.34 to 0.02; *p* = 0.078) (Appendix B).

### 3.10. Metaregressions

**Figure 6:**
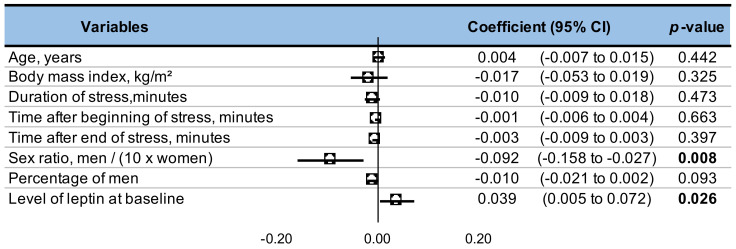
Meta-regression.

Levels of leptin after stress decreased with sex ratio—ratio of males to females (ES = −0.924, 95CI −1.58 to −0.27, *p* = 0.008)—and increased with the baseline levels of leptin (0.039, 0.01 to 0.07, *p* = 0.026). Other clinical characteristics did not significantly influence leptin’s level (age, BMI, percentage of men) and neither did the duration of stress nor the time after the beginning or end of the stress (Figure 6). We also performed a meta-regression on the impact of percentage of women on leptin level: leptin levels increase with percentage of women (ES = 0.01, 95CI 0.004 to 0.016, *p* = 0.008).

## 4. Discussion

Our main findings were that leptin is a biomarker of stress, with a decrease following an acute stress intervention. Moreover, normal-weight individuals and women seem to have a higher response than overweight individuals and men, demonstrating the link between obesity and stress. If other biomarkers of stress are routinely used, leptin should be more studied because of its impact on appetite, obesity genesis, and reproductive function.

### 4.1. Leptin as a Biomarker of Stress

Leptin is a 167-amino-acid peptide discovered in 1994 [37], with the majority produced by the white adipose tissue and the minority produced in other tissues, such as ovary, mammal glands, pituitary gland, skeletal muscle, lymphoid tissue, and stomach, with a prominent effect on the central nervous system [22]. Its receptors are primarily expressed in the hypothalamus and in other brain locations that regulate energy homeostasis and neuroendocrine function [38]. Very interestingly, we demonstrated that leptin is also a biomarker of stress, decreasing after exposure to a stressful environment. Leptin, also named satiety hormone, has two main effects. The first is to stop the food intake with a negative feedback loop [39]. Leptin receptors are mainly located in the arcuate, a part of the hypothalamus that produces pro-opiomelanocortin (POMC) [40]. POMC is a precursor of three main pathways: the melanocyte-stimulating-hormone axis, whose role is to regulate appetite [41] and sexual behaviour [42]; the adrenocorticotropic hormone (ACTH) axis regulating the glucocorticoids secretion mainly implicated in the stress reaction [43]; and the beta-endorphin axis producing endogenous opioids peptides [43]. The decrease of leptin levels after acute stress can be an adaptative mechanism that may help people respond to stress. Indeed, stressful events make it difficult to stay focused on a possibly intense task that could need a great deal of energy. The decrease in leptin levels could induce the signal to increase the food intake in the case that the situation will occur again. Secondly, leptin has a positive effect on energy expenditure [44], with a paracrine pathway in WAT that could be part of the fat-reducing activity of leptin and thermogenesis via the leptin receptor skeletal activation muscle [45]. The sensitivity analysis performed by the exclusion of studies outside of the funnel plot confirm this result. Indeed, funnel plots were first described in 1984 to assess the quality of articles [46]. It is primarily used as a visual aid for detecting bias or systematic heterogeneity [46]. Studies outside of the funnel plot seem to have non-comparable population with the majority of those included in the meta-analysis [46].

### 4.2. Acute Response to an Acute Stress Intervention

We demonstrated that leptin levels decreased in response to acute stress, mainly due to its impact on HPA axis. Therefore, leptin can be considered a biomarker of stress, such as catecholamines [47], heart rate variability [48], cytokines [49], ghrelin [50], or DHEA [51]. Moreover, leptin’s main action is on the hypothalamic-pituitary-axis by increasing POMC, the precursor of glucocorticoids. The strong relationship between leptin and corticoids has been proven with the delayed (6 h) but long-lasting increase in serum leptin (over 16 h) after a single bolus of dexamethasone given before a single large meal [52]. Furthermore, leptin has circadian variations, with the lowest concentration during the day and a higher concentration during the night. More specifically, it has been shown that zenith at midnight and nadir between 9 a.m. and 12 p.m. helps maintain sleep by inhibiting food intake behaviour [53]. Circadian rhythm can be modified by age and BMI [54]. Interestingly, leptin levels need time to decrease, with the lowest levels more than 1 h after the acute stress. It could be explained by its half-life of around 30 min, whereas other biomarkers with a shorter half-life decrease sooner [50]. As no studies assessed leptin levels more than two hours after the acute stress, the duration of the effects of the acute stress on leptin are still unknown. The relationship between obesity and stress is so strong that proposals for international recommendations suggest the implementation of stress management programs in obesity for sustainable weight loss [55,56]. Our study’s benefit is the proof that acute stress decreases leptin levels and thus promotes food intake and may be a pathway towards obesity.

### 4.3. Impact of the Body Mass Index on Leptin

We demonstrated a higher effect on leptin levels in the normal-weight population compared to the overweight people. A possible explanation is the leptin resistance in overweight individuals [57]. This hypothesis is strengthened by our meta-regressions. Indeed, we found a significant impact of a high baseline level of leptin, which is found in overweight and obese individuals [58]. Even if genetic predispositions to diet-induced obesity exist, it is the 24/7 availability of a highly palatable diet that drives overeating [59]. High food intake increases triglyceride production and therefore the WAT production and the leptin levels (leptin being produced by WAT). The vicious circle continues with the apparition of cellular leptin resistance [57]. To achieve this, circulating leptin will rise to create a new equilibrium after expanding the fat mass. The increase of leptin levels in overweight people is associated with diminished leptin transport across the brain–blood barrier. An inhibitory negative feedback appears leading to diminished leptin receptor signal. The increase of free fatty acids and chronic overnutrition seem to induce lipotoxicity and endoplasmic reticulum stress. This inflammatory response might contribute to a blunted physiological response to leptin in obesity [60] even if overweight individuals have a higher circulating leptin level [61] throughout the circadian rhythm [62]. However, the daily variations in overweight individuals are smaller than those in normal-weight individuals. The amplitude as well as the average 24-h leptin concentration were increased by 280% and 420%, respectively, in obese compared to normal-weight women [17]. Furthermore, many genetic models have proven that the leptin gene’s absence induces obesity in animals [60] and in humans [63]. For more than five decades, the energy balance equation had a significant impact on the obesity pandemic. Physical activity decreased drastically, whereas food quantity and availability of high caloric food increased [57]. Initiation of food intake is mediated via ghrelin, insulin, and cannabinoid (36). The main action of leptin arrives after the meal initiation and is responsible of satiety [22].

Unfortunately, the number of studies limited comparisons to two groups (BMI > 25 vs. BMI < 25 kg/m^2^)—insufficient data precluded to further divide the BMI > 25 group into overweight and obese individuals. Moreover, most consequences of fat accumulation begin with a BMI > 25, such as an increased risk of type II diabetes, all cancers except oesophageal (female), pancreatic and prostate cancer, all cardiovascular diseases (except congestive heart failure), asthma, gallbladder disease, osteoarthritis, and chronic back pain [64]. If we did not find any impact of BMI on meta-regression, stratification by weight, i.e., overweight versus normal-weight individuals, showed a significant decrease in normal-weight individuals compared to overweight individuals. The influence of BMI on the relationship between stress and leptin may not be linear. Exploring this relationship would require more data to find some possible threshold. Our study also lacked underweight participants to demonstrate possible U curve.

### 4.4. Impact of Sex on Leptin Levels

Very interestingly, we demonstrated a higher variation of leptin levels in women compared to men after acute stress. Men and women do not have the same WAT repartition. Women had a significantly lower visceral adipose tissue volume than men but a more significant subcutaneous adipose tissue [65]. Studies showed that subcutaneous fat tissue produced more leptin than visceral fat tissue [57]. Secondly, the study of circadian misalignment in shift workers showed that females had lower 24-h leptin levels, while males had higher 24-h leptin levels when misaligned [66]. Those results could promote the implication of leptin in the development of obesity in female shift workers. Furthermore, leptin has also an impact on reproduction, mainly because of the energy state of the body. Sufficient levels of leptin are a pre-requisite for the reproductive capacity in all aspects (regulation of gonadotrophs secretion, ovary function, preimplantation embryo, implantation, placentation, pregnancy, and foetal development). Furthermore, at the ovary level, leptin antagonizes the effect of growth factors on gonadotropin-stimulated steroidogenesis to augment the reproductive function of females [67]. Leptin also seems to be involved on pathogenesis of endometriosis. Indeed, even if there is no difference in serum and plasma, a higher leptin concentration level in peritoneal fluid has been showed in women with endometriosis compared to control. This should induce a local impact of leptin on endometriosis [68]. The link between sex steroids hormones and leptin is strong. Indeed, they are involved in the regulation of leptin transcription, protein secretion in adipocytes, and sexual dimorphism. Amenorrhea can result from a too-low leptin level due to a severe loss of weight. It is a possible adaptive response to low subcutaneous fat to support pregnancy and lactation [69]. Additionally, oestradiol treatment resulted in increased leptin levels, suggesting a role for this steroid in sexual dimorphism. It suggests the higher ratio of oestrogen/androgen is in part responsible for higher leptin levels observed in females compared to males [70]. Lastly, higher circulating leptin concentrations and/or elevated expression of leptin receptors in tumours may be poor prognostic factors [71]. Because of its impact on inflammation, oxidative stress, cell proliferation, inhibition of apoptosis, angiogenesis and immune modulation, leptin seems to impact the development of cancer [71] and specially of breast cancer in obese premenopausal women [72].

### 4.5. Limitations

Our study has some limitations. Meta-analyses inherit the limitations of the individual studies of which they are composed and therefore are subjected to the bias of included studies. Another limitation is related to the publication bias. Indeed, studies with positive effects are more likely published than those with negative effects. However, the use of broader keywords in the search strategy limits the number of missing studies. Only seven monocentric studies were available for meta-analysis, and the total number of subjects included was not as extensive as one would prefer. No included studies had a randomized controlled design. Moreover, blinding of the stress interventions were deemed not feasible. Though there were similarities between the inclusion criteria, they were not identical. Limiting our meta-analysis to studies sharing precisely the same inclusion and exclusion criteria was impossible due to limited data. All studies were monocentric, limiting the generalizability of our results. However, our selection of articles was rigorous, and few studies were outliers when considering metafunnels. The high I^2^ attested heterogeneity between studies. Nevertheless, after the exclusion of studies outside the metafunnel, our results stayed statistically significant. Even if leptin levels follow a circadian rhythm, and time of procedure differs between studies, our meta-analysis is still valid, as each participant is his own control (comparison of leptin levels after the stress to before the stress) within a limited period of time (90 maximum after the stress). Due to the lack of information, we failed to perform meta regressions on the impact of smoking, physical activity, insulin resistance, or levels of cholesterol (LDL, HDL, triglycerides). Unfortunately, the low number of studies (one) precluded analysis on physical stress. After exclusion of studies outside of the metafunnel, we failed to find a significant decrease of leptin levels stratified by weight, mainly because of the weak number of groups (19 versus 28 before exclusion of metafunnel) and only seven in the group “overweight” versus eleven. However, tendency is strong in overweight group, with a *p* = 0.056.

## 5. Conclusions

Leptin is a biomarker of stress, with a decrease following an acute stress intervention. Normal-weight individuals have a higher response, emphasizing the link between stress, obesity, and leptin resistance. Women also have a higher variation of leptin levels after an acute stress. Our findings suggests that leptin may have implications in obesity development in response to stress in a sex-dependent manner. The link between appetite regulation and stress warrants further investigation, and better understanding may be used as a component of weight management strategies. The responses to acute stressful events may provide a novel therapeutic strategy to modify risk factors of obesity development in the hope of improving stress vulnerability, especially in the female population. Furthermore, it could be relevant to study longer or repeated stress to be more related to real-life events.

## Figures and Tables

**Table 1:** Characteristics of included studies.

Study	Country	Study Design	Population			Stress		Leptin Assessment		
			Characteristics	Men	Women	Type	Duration	Time after Stress	Fluid	Technique
				*n*	*n*		(Minutes)	(Minutes)		
Brydon 2008	UK	Cohort	Healthy women	0	67	Mental	10	2 measures: 0 and 45	Blood	ELISA
Caslin 2015	USA	Cohort	Healthy normal and overweight men	20	0	Mental	20	2 measures: 30 and 120	Blood	ELISA
Huang 2014	USA	Case control	Healthy normal and overweight men	20	0	Mental	20	2 measures: 0 and 60	Blood	ELISA
Jones 2016	USA	Cohort	Healthy insulin-resistant and insulin-sensitive volunteers	0	60	Mental (TSST)	13	3 measures: 0, 15, and 90	Blood	RIA
Pop 2010	Romania	Case control	Healthy normal and overweight volunteers	42	37	Physical (VO_2_ max)	12.5	1 measure: 30	Blood	ELISA
Potrezke 2014	USA	Case control	Healthy smokers (relapsers and abstainers)	22	14	Mental	24	4 measures: 0, 30, 46, and 76	Blood	ELISA
Tomiyama 2012	USA	Cohort	Healthy postmenopausal women	0	40	Mental (TSST)	10	3 measures 0, 50, and 90	Blood	ELISA

**Figure A1:**
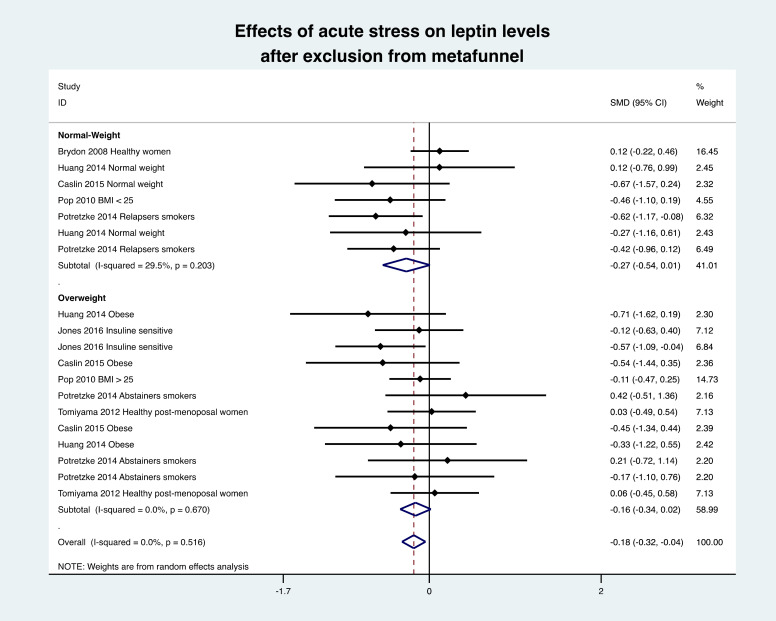
Meta-analysis of leptin levels after an acute stress compared to baseline levels after exclusion of studies outside of the metafunnel.

## Data Availability

Data sharing not applicable.

## Appendix A

Details for the search strategy used within each database.

*PubMed*
(“leptin”[MH] OR “ob protein”[TW] OR “ob gene product”[TW] OR “obesity factor”[TW] OR “obese gene product”[TW] OR “obese protein”[TW] OR “leptin”[TW])
AND (“Stress, Psychological”[Mesh] OR “Anxiety”[Mesh:NoExp] OR anxie*[TIAB] OR anxious*[TIAB] OR stress*[TIAB] OR mood[TIAB] OR emotion[TIAB])
Filter Language = none
Filter Dates = none

*Web of science (WOS)*
(leptin OR “ob protein” OR “ob gene product” OR “obesity factor” OR “obese gene product” OR “obese protein”)
AND (anxie* OR anxious* OR stress* OR mood OR emotion)
Filter Language = none
Filter Dates = none

*Cochrane Library*
(leptin OR “ob protein” OR “ob gene product” OR “obesity factor” OR “obese gene product” OR “obese protein”)
AND (anxie* OR anxious* OR stress* OR mood OR emotion)
Filter Language = none
Filter Dates = none

*Embase*
(leptin OR “ob protein” OR “ob gene product” OR “obesity factor” OR “obese gene product” OR “obese protein”)
AND (anxie* OR anxious* OR stress* OR mood OR emotion)
Filter Language = none
Filter Dates = none
